# Benefits of Integrating Technology into Home Exercise Therapy in Patients with Lower Extremity Peripheral Artery Disease

**DOI:** 10.3390/jcm12247635

**Published:** 2023-12-12

**Authors:** Andreea Rotundu, Andra Oancea, Alexandra Maștaleru, Alexandru-Dan Costache, Carmen Marinela Cumpăt, Irina Mihaela Abdulan, Anisia Iuliana Alexa, Costin Chirica, Mara Russu, Maria Magdalena Leon

**Affiliations:** 1 Doctoral School, “Grigore T. Popa” University of Medicine and Pharmacy, 16 Universității Str., 700115 Iasi, Romania; rotundu.andreea@d.umfiasi.ro (A.R.); chirica.costin@d.umfiasi.ro (C.C.); mararussu@yahoo.com (M.R.); 2 Department of Medical Specialties I, “Grigore T. Popa” University of Medicine and Pharmacy, 700115 Iasi, Romania; dan-alexandru.costache@umfiasi.ro (A.-D.C.); irina.abdulan@umfiasi.ro (I.M.A.); maria.leon@umfiasi.ro (M.M.L.); 3 Clinical Rehabilitation Hospital, 700661 Iasi, Romania; marinela.cumpat@umfiasi.ro; 4 Department of Medical Specialties III, “Grigore T. Popa” University of Medicine and Pharmacy, 700115 Iasi, Romania; 5 Department of Surgery II, Discipline of Ophthalmology, “Grigore T. Popa” University of Medicine and Pharmacy, 700115 Iasi, Romania; anisia-iuliana.alexa@umfiasi.ro

**Keywords:** peripheral arterial disease, telerehabilitation, home-based exercise

## Abstract

(1) Background: Telerehabilitation is an approach that uses digital technology to provide remote medical recovery services. It can be an option for cardiovascular recovery at home in patients with peripheral arterial disease (PAD) of the lower limbs. (2) Methods: We performed literature research through two databases: PubMed and Embase. We included randomized controlled trials and cohort studies that evaluated the effectiveness of a technology-assisted home exercise intervention compared with conventional rehabilitation or the usual care in patients with PAD. We analyzed population, intervention, and outcome data. (3) Results: We identified 2468 studies. After rigorous screening, we included 25 articles in the review. The following results were evaluated: dissemination and acceptance of digital technologies among these people, functional capacity, exercise intensity, patient motivation, sex-specific response differences in mortality and clinical outcomes, quality of life assessment, and changes in values of inflammatory biomarkers. All of these were correlated with the type of intervention and the dose of the exercise. (4) Conclusions: Home-based exercise therapy supervised with the help of specific devices could be successfully implemented in the therapeutic management of the PAD population. Health specialists should take into account the clinical–paraclinical profile and the emotional status of the patients. Such individualized interventions could bring significant benefits for the people with this disease and for the healthcare system, including increasing exercise adherence, engagement, self-care capacity, life expectancy, and quality of life for these patients, as well as reducing their symptoms, cardiovascular complications, and hospitalizations.

## 1. Introduction

Cardiovascular diseases (CVDs) rank among the primary contributors to global mortality. Despite notable technological progress in the diagnostic and therapeutic approach to this pathology, the mortality rate continues to rise, having increased by 6.5 million between 1990 and 2019 [1]. Peripheral artery disease (PAD) is the third most prevalent cause of atherosclerotic diseases, following coronary artery disease (CAD) and stroke [2]. Currently, data from epidemiological studies show that approximately 236 million adults are diagnosed with PAD [3]. The main symptom of this pathology is intermittent claudication (IC), defined as calf pain or discomfort caused by exertion, which improves with rest [4]. Thus, patients are limited in their routine activity. It results in a significant decline in the overall quality of life, with more frequent hospitalizations and prescribed treatments, thus burdening the healthcare system [5,6].

According to the guidelines provided by the European Society of Cardiology for the management of patients with PAD, it is recommended that individuals affected by this condition participate in and adhere to a cardiac rehabilitation (CR) program [7]. This recommendation is supported by extensive clinical trials that have demonstrated significant improvement in maximal walking distance without claudication in individuals with PAD through exercise training [8]. CR can be performed both in a specialized rehabilitation center and at home. Recent studies have shown similar walking perimeter benefits in both situations [9]. Moreover, it is recommended that patients start the CR program in a specialized rehabilitation center. Here, the patient benefits from a comprehensive evaluation in order to prescribe individualized exercise training [7].

Supervised exercise therapy (SET) has been shown to improve maximal oxygen consumption, increasing walking distance without experiencing calf pain, leading to better socioeconomic integration [10]. Despite its benefits, this approach has a poor adherence rate due to the long duration of the hospitalization and a reduced number of specialized medical centers [11]. Moreover, to maintain these results, the patient should continue the training for a long time, with periodic follow-ups. Accomplishing these outcomes can be challenging, particularly for elderly patients with restricted mobility. Consequently, guidelines offer an alternative approach, allowing exercises to be conducted within the home environment. However, the drawback of this method is the absence of mechanisms for monitoring and guiding these patients [12].

Recent studies, which included patients who performed exercises at home under the supervision of a specialist, demonstrated definitive benefits in improving symptoms, reducing depression and anxiety, as well as mortality. All participants were initially evaluated in order to establish the indications regarding the intensity and type of training. The supervision was carried out with the help of specific devices, which allowed the quantification of the outcomes. Data from this research showed no statistically significant differences in symptomatology comparing the exercises of the two types of rehabilitation [13].

Evidence-based approaches involving the use of devices such as mobile phones and smartwatches in exercise interventions demonstrated significant benefits in patients with PAD [14]. The mobile health approach facilitates extensive surveillance and patient monitoring without involving many human resources [15]. Technology-based interventions allow healthcare providers to issue real-time therapy advice. They also monitor symptoms and potential cardiovascular events without location restrictions [16]. Thus, well-designed technology-based interventions can be used to provide health education, promote behavioral change, and have the potential to improve exercise adherence [17].

Telerehabilitation is an innovation in the field of CR through which patients receive indications of physical exercises according to PAD staging. Their evolution is followed with the help of the devices by the attending physician. Telerehabilitation could represent an easier way to carry out physical training and follow the evolution of patients. The benefits are a faster social and family reintegration of people with PAD, a decrease in anxiety and depression dependent on hospitalization, and the improvement of self-care capacity. However, the data from specialized literature on its application are limited.

Previous reviews on patients with PAD have focused mainly on the effects of SET and endovascular revascularization [18]. Some studies have attempted to identify more effective screening methods for PAD and factors that influence adherence to physical activity. In contrast, existing evaluations have not analyzed the effects of device-supervised home exercise interventions on patients with PAD. In order to guide the development of telerehabilitation in the management of people with PAD, scientific evidence is needed regarding exercises performed at home and their remote monitoring through technology.

Thus, in this study, we performed a review with a particular emphasis on the benefits of home exercises monitored with technological devices. We focused on improving functional capacity, establishing exercise intensity, increasing motivation and quality of life, reducing inflammation, and decreasing mortality rates in PAD patients.

## 2. Materials and Methods

The review question was: “What benefits does integrating technology into home exercise therapy bring to patients with PAD?”

The population studied in this research consisted of adults with PAD. The intervention was home-based exercise rehabilitation monitored from a distance by the instrumentality of technological means in PAD patients. The control group consisted of the usual care or supervised cardiac rehabilitation. The results were: dissemination and acceptance of digital technologies among these people, functional capacity, exercise intensity, patient motivation, sex-specific response differences in mortality and clinical outcomes, quality of life assessment, and changes in inflammatory marker values.

### 2.1. Search Strategy

We indexed the relevant publications from the Embase and PubMed databases after a structured search based on the following formula: (‘peripheral arterial disease’/exp or ‘peripheral arterial disease’ or (peripheral and arterial and (‘disease’/exp or disease)) or ‘peripheral artery disease’/exp or ‘peripheral artery disease’ or (peripheral and (‘artery’/exp or artery) and (‘disease’/exp or disease)) or pad or ‘lower extremity arterial disease’/exp or ‘lower extremity arterial disease’ or (lower and (‘extremity’/exp or extremity) and arterial and (‘disease’/exp or disease)) or ‘lead’/exp or lead or ‘peripheral vascular disease’/exp or ‘peripheral vascular disease’ or (peripheral and vascular and (‘disease’/exp or disease)) or pvd or ‘intermittent claudication’/exp or ‘intermittent claudication’ or (intermittent and (‘claudication’/exp or claudication)) or ic) and (‘telemedicine’/exp or telemedicine or ‘telerehabilitation’/exp or telerehabilitation or ‘technology’/exp or technology or ‘activity monitoring interventions’ or ((‘activity’/exp or activity) and (‘monitoring’/exp or monitoring) and (‘interventions’/exp or interventions)) or ‘mobile health home-based training’ or (mobile and (‘health’/exp or health) and ‘home based’ and (‘training’/exp or training)) or ‘walking program’ or ((‘walking’/exp or walking) and program) or ‘home-based exercise’ or (‘home based’ and (‘exercise’/exp or exercise))).

### 2.2. Study Selection

We considered eligible for our narrative review studies that evaluated an intervention based on home-based exercise therapy delivered through new technologies. The benefits of this therapeutic strategy were assessed by comparing the intervention group with the usual care group. We applied the following selection criteria:

- Study type: randomized controlled trials, cohort studies;
- Language: English;
- Year of publication: 2013–2023;
- Types of participants: people over 18 years old who have been diagnosed with PAD;
- Types of interventions: home exercise, a walking program, activity monitoring interventions, home mobile health training telemedicine, telerehabilitation, individual and group-mediated cognitive–behavioral interventions and gamification;
- Outcome: the dissemination and acceptance of digital technologies among the PAD population, patients’ functional capacity, motivation and quality of life, exercise intensity, sex-specific response differences in mortality and clinical outcomes, and post-therapeutic changes in inflammatory marker values;
- Follow-up duration: without restrictions.

We established some inclusion criteria: (1) cohorts were composed of adults with PAD; (2) patients performed exercise interventions at home, supervised from a distance by authorized personnel with the help of devices (mobile phones, smartwatches); (3) the results were relevant to the study design; (4) the study design was presented.

We excluded the following studies: (1) the articles were not original; (2) they were not published in English; (3) not all patients underwent the same treatment; (4) rehabilitation techniques have not been fully described.

**Figure 1:**
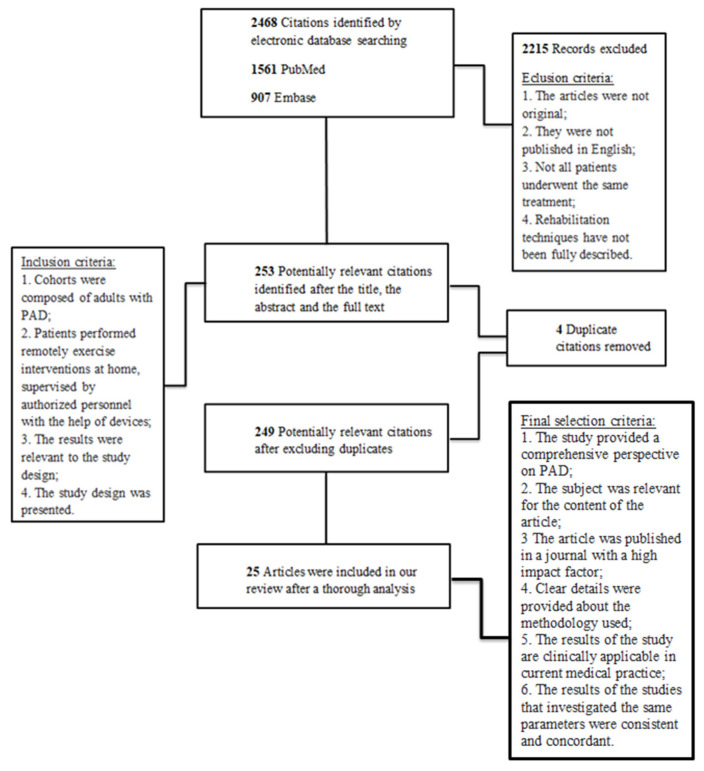
Study selection. PAD: Peripheral artery disease.

The study selection method is illustrated in Figure 1.

### 2.3. Study Analysis

After a thorough analysis of the title, the abstract, and the full text of 2468 studies, we included in our review the most relevant 25 articles.

### 2.4. Data Extraction

We extracted the following data from the studies: author, year of publication, place of study enrollment, number of participants, population characteristics (age, gender, education level), and follow-up time. We also took into account the following details about the interventions: the type and description of the intervention (number of sessions, duration of each session in minutes, number of steps/sessions, speed and intensity of the exercises) and the comparator. All the results were reported along with the measurement method.

## 3. Results

### 3.1. Dissemination and Acceptance of Digital Technologies among Patients with PAD

The evolution of technology has brought significant benefits to current medicine, enabling the improvement of remote communication between doctors and patients through telemedicine. At the same time, the new technological means allow patients to self-monitor their vital parameters (blood pressure, heart rate) as well as their progress in terms of functional capacity (daily number of steps, walking distance, walking time). However, there are a limited number of studies that evaluate the use of technology among patients with PAD.

This pathology is known to be more common in elderly people. This is also supported by a study of 3.6 million patients, which demonstrated that the prevalence of PAD increases with age, ranging from 1.2% in the 40–50 age group and reaching 24.1% for patients aged 91–100 years [19]. Recently, researchers suggested that especially older patients are doubtful about accepting and using these devices.

Alushi K et al. included in their study 326 participants aged between 60 and 84 years. Only 66.8% of them owned a smartphone. Several factors negatively influenced the acceptance and use of digital technology among the study population, such as older age, the association of intermittent claudication, lower educational status, living in rural areas, the need for support in using apps and smartphones, and the preference for television as a source of health information. These aspects suggest that the use of mobile health applications must be individualized to patients to avoid missing an important subpopulation of subjects with PAD [20].

On the other hand, a study based on a telemedicine program (Control Telehealth Claudication Intermittent (CONTECI)) showed that using digital technology in the care of the PAD population can play an essential role in obtaining better outcomes. The intervention aimed to train patients to be able to understand their health problems and self-monitor their disease. Achieving this goal has led to increased treatment compliance (at 6 months, treatment adherence improved by 83.8% in patients in the intervention arm, compared to 69% in those in the control arm, but this was not maintained at 12 months), early recognition of complications (7.85 days faster in the intervention group, compared to 53.89 days in the control group), and improved satisfaction and quality of life (improvement in the patient satisfaction evaluation score ranged from 67.36% to 76.78%, and the quality of life assessment score from 67.87% to 72.25% in the intervention group between baseline and the 12-month assessment), while reducing the use of medical resources (decrease in the number of scheduled medical visits by 95.95% in the intervention group). This study demonstrates that self-management of health through digital technology is feasible for PAD patients [21]. Another study based on monitoring the number of steps with the help of a pedometer and telephone educational sessions showed that the patients appreciated participating in this type of intervention. Moreover, they had a better understanding of their health status and became more aware of their disease compared to the time of enrollment. Furthermore, they could better follow the individualized prescription rehab program in a more feasible way using the pedometer [22].

### 3.2. Improving Functional Capacity

Previous studies have demonstrated that patients diagnosed with PAD exhibit a lower number of steps per day compared to patients without this disease [23]. The lack of physical activity leads to PAD progression, disability, and cardiovascular events [24]. Researchers consider exercise therapy as being one of the main treatment methods for patients with PAD, as it has been proven to improve functional capacity [7,25,26].

McDermott et al. performed a study that included 200 participants with PAD who had their home exercise intervention evaluated and guided by electronic devices and telephone coaching. The intervention group consisted of 99 patients, and it was compared to a control group of 101 participants who did not receive any exercise prescription. The authors remarked that this intervention did not correlate to a significant improvement in the 6 min walk distance (6-MWD) test results at the 9-month follow-up. Additionally, at the end of this period, patients were evaluated by the Walking Impairment Questionnaire (WIQ), by the physical functionality score short form (Medical Outcomes Short Form 36-SF-36), by the score of measuring the results reported by the patient (Patient-Reported Outcomes Measurement Information System—PROMIS) and by the objective measurement of change in physical activity. The results indicated that the intervention worsened the PROMIS pain assessment. Also, there were no statistically significant improvements in WIQ and SF-36 scores in the home exercise group compared to the non-exercise group [27].

Recent studies have demonstrated the benefits of home-based exercises in terms of claudication onset time (COT) [28,29,30], maximal walking speed (S_max_) [31,32], and 6-MWD [22,30,33,34]. Also, this therapy increased the peak walking time (PWT) [29,30], and the patients reported better results of SF-36 [35] and WIQ [22,28,33,34,36]. All this evidence supports the use of therapy based on physical exercises at home as a strategy to improve functional capacity in this category of patients (Table 1).

### 3.3. The Effects of Home-Based Exercise Intensity

McDermott et al. performed a study on 305 participants diagnosed with PAD that were divided into three groups: two of them were prescribed different types of activity and were divided according to the intensity of the exercise. The third control group did no training. Walking at low intensity was defined as the rhythm of the pace that did not induce IC, while patients undergoing high-intensity training experienced this type of complaint. At a 12-month follow-up, exercises that induced ischemic symptoms resulted in a better improvement in the 6-MWD compared to the other group. However, no statistically significant difference has been found comparing the low-intensity exercise group with the control group regarding the 6-MWD [33].

Further analysis of the same study showed that people with PAD who performed walking exercises at a higher intensity increased their walking speed (assessed by walking velocity over 4 m) at the six-month follow-up, with a speed of 0.056 m/s, and at the twelve-month follow-up, with 0.084 m/s, and had an improved Short Physical Performance Battery (SPPB) score at the twelve-month follow-up, with a score of 0.821, compared to people who performed walking exercises at a low intensity. Compared with a no-exercise control group, home walking exercise at an IC-inducing pace significantly improved walking speed by 4 m (at 0.066 m/s) after months of intervention. However, at the one-year examination, these results were no longer maintained. Therefore, high-intensity walking is more beneficial for PAD patients than more frequent or longer walking, which encourages us to recommend the issuance of prescriptions based on IC-inducing home exercises in PAD patients [37].

Another study based on a home-based rehabilitation program for PAD patients of moderate intensity (intermittent walking until mild to moderate IC occurs) showed significant improvements in pain threshold velocity (PTS), S_max_, PFWD, 6-MWD, ankle-brachial index (ABI) and increased adherence. Thus, pain-free, moderate-intensity walking sessions with interval breaks were accepted and successfully followed by these patients [31].

Atherosclerotic diseases, including PAD, are characterized by autonomic dysfunction [38], which can be defined as reduced heart rate variability (HRV) [39]. A study investigated the effects of a 3-month home walking program that did not induce ischemic calf symptoms on circulatory and autonomic function in PAD patients with IC. The findings of the study indicated that undergoing pain-free exercises can lead to improvements in central autonomic function, as evidenced by optimal HRV control. This optimization may also have potential benefits for peripheral autonomic adaptation. A higher parasympathetic activity associated with a decreased activity of the sympathetic system observed in the study leads to a reduction in the risk of cardiac morbidity and mortality. Furthermore, the diminution of sympathetic activity during exercise can improve blood flow to the lower limbs, leading to improved walking ability. These findings highlight the potential of low-intensity exercise as a therapeutic approach to improve autonomic function and cardiovascular health in patients with symptomatic PAD [40].

These findings emphasize the importance of communication between patients and their cardiac rehabilitation team in order to determine the appropriate individualized intensity of exercise. This personalized approach can maximize the benefits by reducing the risk of exacerbating symptoms or experiencing complications.

### 3.4. Patient Motivation Techniques to Improve Outcomes

Mobile technologies have shown promise in promoting health behavior changes, but the challenge lies in sustaining these changes over time. One limitation of remote monitoring through mobile technologies is the potential lack of patient engagement and motivation. While mobile applications for physical activity and dietary behavior change exist, it is essential to assess whether they incorporate evidence-based behavior change techniques. Additionally, health behavior models need to be adapted to the context of mobile interventions to ensure their effectiveness. Therefore, while mobile technologies offer opportunities for promoting health behavior change, addressing the issue of long-term maintenance and patient engagement remains a significant challenge that requires further research and development.

Gamification is a concept of modern medicine that involves taking elements from games and integrating them into training and follow-up strategies to increase patient engagement and motivation. New technological devices make these things possible by recording, collecting, and quantifying people’s daily physical activity and displaying real-time progress. Any physical effort that can be rewarded with instant positive feedback from medical staff while walking is a real win for PAD patients rehabilitating at home. In this way, they are encouraged to increase their number of steps despite the discomfort and barriers. A pilot study, which used all of these tools to increase patient motivation, evaluated whether this new approach improved daily activity and adherence of people with PAD to home exercise programs. Feedback-based home activity monitoring was associated with increased 6-MWDs, daily steps, engagement, satisfaction, and patient compliance. This type of intervention has been shown to be more beneficial for individuals with lower baseline activity [41].

Both individual and group-mediated cognitive–behavioral interventions may be an acceptable option for increasing the motivation of these patients.

In the Motivating Structured Walking Activity in People with Intermittent Claudication (MOSAIC) study, 190 patients diagnosed with PAD and experiencing IC were divided into two groups. The intervention group performed walking exercises at home and participated in individual sessions to support health-related behavior change. The control group received only standard care provided by vascular specialists. The authors found that the group receiving individual cognitive–behavioral intervention for home walking exercise showed significant improvement in the 6-MWD at three months compared to the control group. This aspect can be attributed to several factors, including psychology, such as positive walking beliefs, a correct understanding of the pathology, and the ability to control the disease. All of these could have contributed to increased motivation among the participants. Additionally, the intervention was individualized according to the intellectual level of each participant, the functional capacity, and the background, which likely facilitated health behavior change and turned the desire to walk into real fact. Another important finding of this study was that the exercise and educational therapy group had a lower risk of mortality compared to the control group. This further supports that this therapeutic strategy makes an essential contribution regarding both functional capacity and general health in people with PAD [42].

The Group Oriented Arterial Leg Study (GOALS) included 194 people with PAD with and without IC. The researchers tested the effects on functional capacity among patients who followed a program based on home walking exercises and group-mediated cognitive–behavioral therapy. The control group attended only PAD health education sessions and did not exercise. The weekly group-mediated cognitive–behavioral therapy sessions involved expert-moderated discussions on topics such as the benefits of walking exercise for PAD, goal-setting, self-monitoring, and pain management during exercise. All of this was aimed to increase patients’ motivation and ability to self-manage the disease. Following this program, the 6-MWD, WIQ score, and the physical and mental health composite scores from the Medical Outcomes Study 12-Item Short-Form Health Survey (SF-12) improved significantly in the participants of mediated cognitive–behavioral intervention compared to those in the other group [34].

Another study evaluated the effectiveness of a program that involved group-mediated cognitive–behavioral therapy and home exercises on preventing loss of mobility and increasing functional performance compared to only participating in health education sessions. The program consisted of two consecutive phases, each lasting six months. After completing the two phases, the following were reported: significant improvement in the intervention group compared to the control group in fast-paced 4 m walking speed at a 6-month follow-up (1.24 to 1.29 m/s vs. 1.22 to 1.19 m/s), the cumulative rates of regaining mobility among participants with loss of mobility at baseline at 6 and 12 months follow-up improved (82.6% vs. 36.4%), and the SPPB physical performance scores had improved at a 12-month follow-up (9.87 to 10.33 vs. 9.93 to 9.81) [43].

All these findings underline that promoting the motivation of PAD patients is essential to ensure their long-term involvement and engagement. Motivation can be successfully achieved through numerous means.

### 3.5. Gender-Specific Response Differences in Mortality and Clinical Outcomes

A cohort study assessed sex-specific response differences in mortality rate, risk of all-cause acute hospitalizations, and number of major and minor amputations over seven years between a group that followed a home exercise program and a control group that did not perform training. The study provided evidence that patients enrolled in a home rehabilitation program exhibit a higher survival rate (90% in female group and 82% in male group) compared to those receiving the usual care (45% in the female group and 44% in the male group). Furthermore, in the home exercise group, women had a higher survival rate (90%) than men (82%). In addition, in comparison to the control group, the group of patients who underwent rehabilitation demonstrated lower rates of long-term hospitalization (88% for women and 89% for men, compared with 66% for women and 68% for men) and amputations (4% for women and 10% for men, compared with 3% for women and 3% for men) [44].

Considering a series of findings of some previous studies according to which women had a lower basic functional capacity, the lack of reaching the primary objectives in terms of functional capacity can also be a problem specific to the female gender [3]. In addition, weaker improvements in functional outcomes such as COT and PWT have been reported in women with PAD and diabetes after exercise therapy [45]. One study examined whether the baseline clinical profile, duration, and walking intensity during a 3-month pedometer-based home exercise program influenced COT, PWT, and 6-MWD in symptomatic patients with PAD. The results of this study showed that a faster ambulatory cadence had favorable effects on functional capacity in women, while in men, less severe PAD and fewer comorbidities at baseline were associated with improved ambulatory function [30].

A single-center retrospective cohort study evaluated whether functional outcomes differ by gender after a home-based rehabilitation program for people with PAD, based on a prescription of structured, personalized, moderate-intensity exercise. At baseline, there were no gender differences in PAD severity, but the female group, compared to the male group, had poorer results on the 6 min treadmill walking test, PTS, Smax, PFWD, and 6-MWD. Despite these initial differences, performing pain-free exercise sessions has been shown to be equally effective in men and women in terms of improving adherence, functional capacity, and hemodynamic constants. These findings underline the importance of creating programs adapted to women’s needs, with special attention to the intensity, duration, and tolerability of the exercise. In this program, the training modality allowed all exercises to be performed comfortably at home, without giving up family supervision or housework, which are typical duties of most women. The time allocated to the small number of exercises was short and fixed, giving females the opportunity to manage the rest of the responsibilities. In addition, unlike a supervised program that involves hospital admissions at least three times per week, the current intervention involved short visits, low costs, and negligible family involvement. The program was offered to patients free of charge, with the intervention entailing little expenditure on the part of the healthcare system [31].

The presented data proves that it is important to recognize the specific gender differences of the PAD population. Also, it is essential to adapt the therapy based on physical exercises at home according to the needs and the individual clinical–paraclinical profile of the patients.

### 3.6. The Quality of Life

Some studies have reported a significant improvement in physical status, mobility, social and professional performance, and emotional status in this category of patients. One study measured the effects of integrating an educational therapeutic program into home exercise therapy on QoL (assessed by the SF-36 questionnaire), on the control of cardiovascular risk factors, and on functional capacity. Outcomes were tracked both during the first three months of educational courses and by telephone coaching, as well as on extended periods after the patients were no longer trained. The intervention had positive results on patients’ disease self-management after three months. These results were maintained after 12 months, which may explain the long-term improvement in QoL observed in this study population [35]. Another study showed similar benefits of home exercise therapy on postinterventional QoL (assessed by the Vascular Quality of Life questionnaire) 3 months after a group of PAD patients underwent endovascular treatment [46].

However, there are also studies that did not identify beneficial effects in the QoL in patients with PAD who performed exercises at home. In the MOSAIC study, one of the secondary outcomes was the QoL of patients who underwent an individual intervention to change their walking exercise behavior at home. At a 6-month follow-up, the QoL of patients in the intervention group did not improve compared to the usual care group [42]. Another study, which included 200 participants, compared the effects on QoL (assessed by the SF-36 questionnaire) in the group of patients who followed a home exercise intervention with the control group. In the first month, patients in the intervention group attended weekly walking sessions with a trainer at the medical center. Also, they were helped to set their goals and were shown how to enter their results into the study’s online platform. In the following eight months, they monitored their exercises performed at home with a wearable activity monitor. Then, they reported the results on the study’s website and received instructions through telephone coaching. The control group did not perform training sessions at the medical center or exercise at home and did not receive telephone coaching. The results did not show statistically significant differences between the two groups regarding QoL improvement [27].

The lack of improvement in QoL after these interventions can be primarily attributed to the multiple comorbidities that patients with PAD have, which can also negatively influence their psycho-emotional and functional well-being. Secondly, people may underestimate changes in QoL because results occur over a more extended period of time and are less obvious than other interventions, such as revascularization. Last but not least, the progress in mental health is more challenging to observe and appreciate than physical health.

### 3.7. Changes in Inflammatory Biomarkers

Inflammation plays a significant role in the pathogenesis, onset, and progression of PAD, which is widely recognized as a condition associated with chronic inflammation. However, recent studies investigating the efficacy of home exercise therapy in reducing inflammation among patients with PAD have yielded conflicting findings.

Some studies have shown that home physical therapy can have a beneficial effect on reducing inflammatory markers in these patients. One study evaluated the changes in the inflammatory markers in patients with PAD prescribed with home-based exercise programs. The number of steps taken daily and the duration of the activity were monitored using a wearable device. The high-sensitivity C-reactive protein (hs-CRP) value was negatively correlated with the cadence of the ambulatory activity, the number of daily steps, and the number of minutes spent standing. Therefore, higher daily ambulatory activity is associated with lower levels of hs-CRP [47]. Gardner et al. compared the effects that training has on inflammatory markers in 3 groups of subjects prescribed with different types of activity. During the training sessions, all subjects wore an activity monitor. A group of patients (NEXT Step) performed three weekly sessions of walking exercises at home, monitored remotely. Another group (SET Step) followed a supervised exercise program on the treadmill thrice weekly. In the first two groups, the duration and the intensity of the exercises were progressively increased. The control group did not perform walking exercises, only light upper and lower limb resistance training. The NEXT Step group had the most significant decrease in hs-CRP value (24% compared to the initial value), which supports the result of the previous study, which found that daily exercise at home reduced hs-CRP levels in patients with PAD [29].

Other researchers did not provide similar favorable results regarding the effectiveness of home exercise therapy in reducing inflammatory markers. A study published in the Journal of Vascular Surgery evaluated the changes in IL-6 and TNF-alpha in three groups of patients who performed different training programs for 12 weeks. Patients in the home rehabilitation group performed at least three sessions per week of intermittent walking until mild to moderate claudication pain. The duration and intensity of the exercises for the next month of training were determined based on the results recorded with a portable device, which they handed over to the research staff at the end of weeks 1, 4, 8, and 12. The participants in the supervised physical exercise group performed three weekly sessions of intermittent walking on the treadmill until mild to moderate claudication pain, during which they also wore a pedometer. The on-site specialists progressively increased the intensity and duration of their training. The control group did resistance training three times a week without any walking exercise. None of the three groups showed any statistically significant changes in TNF-alpha and IL-6 values between the initial moment and the end of these interventions [48].

These contradictory results can be attributed to the differences in the duration of the treatment, the intensity of the physical exercises, and the particularities of the patients included in the studies.

## 4. Discussion

In the era of technological advances, digital devices and mobile healthcare applications have become widely available, with an increasing number of users in various domains. The concept of digital health includes mobile applications, wearable devices, and components of telemedicine. All of these have an essential role in providing medical care, centralizing and analyzing patient-reported outcomes, and improving the quality of life of people with CVD. Since the emergence of the coronavirus (COVID-19) pandemic, the use of digital technology in the healthcare system has progressed at an accelerated rate in order to provide better for care needs. Digital technologies based on smartphones and applications can improve the doctor–patient connection and help patients monitor their symptoms, track their progress in terms of functional capacity, receive feedback on it in real time, and participate in educational sessions from a distance.

The adherence to supervised high-intensity exercise therapy in the PAD population is reported to be low despite the recommendation by the current guidelines. Several studies have highlighted this issue and found that many patients struggle to adhere to the prescribed exercise program. One of the factors responsible for this shortcoming is that during exercise, people with this disease may experience pain due to lower limb ischemia [39,49,50]. Moreover, patients with severe forms of PAD and multiple comorbidities are frequently excluded from CR programs due to the impossibility of performing this type of exercise [30]. To reduce these symptoms, patients with PAD could be prescribed individualized exercise, which does not induce ischemic symptoms and may improve their adherence to recovery therapy, but the benefits of these strategies are unknown [51].

The incidence of CVD is known to be different between females and males. The gender-specific hormone levels have both protective and harmful effects on the cardiovascular system. Gender differences influence the onset, course, treatment, and outcomes of CVD, including PAD [3]. For instance, the prevalence of these pathologies in women is lower in the premenopausal period, but it increases in the postmenopausal phase due to the loss of the protective effect of estrogen [52]. Previous studies have shown that typical symptoms of PAD are frequently absent in women, but they experience lower functional status and faster functional decline than men do [53]. Literature data regarding the rate of major cardiovascular events, postoperative outcomes, and short- and long-term mortality in both genders with PAD undergoing surgery is contradictory.

Rehabilitation programs at home are based on performing exercises in an unsupervised setting, which involves the risk of failure to achieve the set objectives. Unlike the supervised programs, the unsupervised ones are carried out without a professional to monitor the patients, adjust the intensity and duration of the exercises according to their tolerance, constantly evaluate their progress, and provide them with feedback. This can make it difficult to identify possible problems and solve them in a timely manner. That is why it is essential to identify the eligible patients for home rehabilitation programs based on a complete medical consultation and an individualized exercise plan. The intensity and duration of the exercises have to be chosen according to the needs and abilities of each individual. Also, correct and regular monitoring is essential to ensure the achievement of the established objectives.

**Figure 2:**
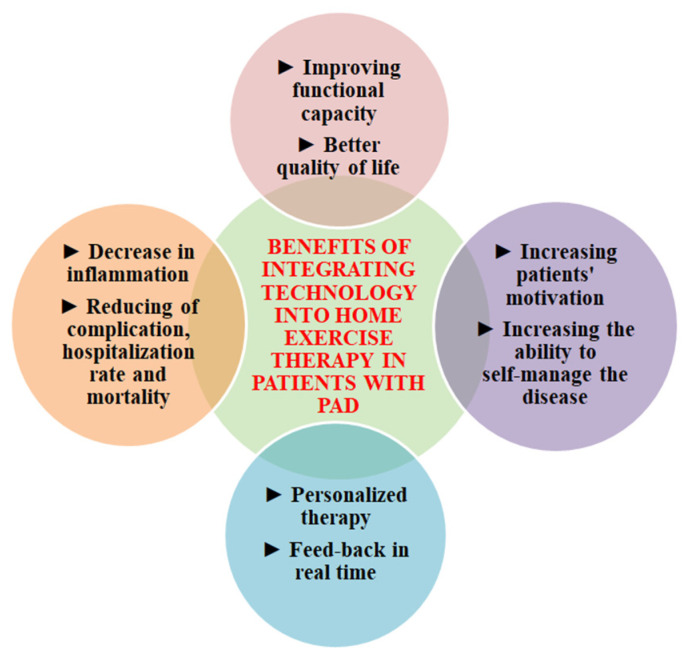
Benefits of integrating technology into home exercise therapy in patients with lower extremity peripheral artery disease (PAD).

The quality of life (QoL) of patients with PAD is severely affected by the symptoms and consequences of this disease. The physical decline of these patients is accelerated by the typical and atypical symptoms of the disease, which limit their mobility. Critical limb ischemia, a severe complication of PAD, is associated with a high risk of amputation. In addition to reduced functional capacity, other factors can negatively influence QoL. Social and professional limitations can often lead to feelings of isolation and exclusion. Also, these patients face difficulties in performing daily tasks such as shopping or personal care. Moreover, this disease affects the emotional state, with patients often experiencing feelings of anxiety, depression, frustration, and loss of independence. To our knowledge, this is the first narrative review that attempts to summarize the benefits of integrating technology into home exercise therapy in patients with PAD. More precisely, the evidence presented is promising in terms of the effectiveness of these interventions in achieving challenging objectives such as increasing walking distance, motivation, and quality of life and reducing symptoms, inflammation, and mortality in the PAD population (Figure 2).

Detailing the interventions from the studies that are part of our analysis (the number of training sessions, their duration, and intensity), we propose a personalized approach to the technologically assisted rehabilitation at home of PAD patients.

In addition, we want to specify that we have not identified any study that evaluates patient safety, the risk of falls, and complications associated with this type of intervention performed in an unsupervised setting and addressed to an elderly population with multiple comorbidities. Future research should also focus on these potential adverse effects that the presented therapeutic strategy implies.

We mention that there were several limitations in our research. Some of the studies considered in our analysis included a limited number of patients, which could affect the generalizability of the results. Also, the exercise protocols and the technologies used varied, which made it difficult to compare the effectiveness of the interventions. Moreover, the lack of a common standard protocol can affect the validation of research. Some studies were based on a poorly defined control group, imposing difficulties in assessing the effectiveness of exercise therapy at home compared to other types of rehabilitation or to the lack of intervention. Furthermore, the long-term effects of this therapeutic strategy are difficult to estimate, considering the short duration of some studies. Additionally, most of the technologies used in exercise therapy are not standardized or validated, which can influence the reliability of the results. Also, devices may have limitations related to measurement accuracy and portability. The heterogeneity of the included studies draws attention to the need to conduct randomized controlled trials of telemedicine interventions based on a detailed implementation methodology.

## 5. Conclusions and Future Directions

In conclusion, the dissemination and acceptance of digital technology among patients with PAD is gradually progressing, and the use of mobile devices and mobile applications can bring significant benefits to the care of these patients. In the era of modern medicine, the integration and use of digital technology among patients with PAD is gradually advancing, offering significant potential for improving disease management. However, it is essential to address potential barriers to ensure its efficacy and safe use for the benefit of patients with this disease. Possible strategies can remove obstacles within the framework of future home exercise interventions monitored through technology. These strategies include facilitating accessibility to technology, educating and training patients, continuous support, personalization and adaptation of devices according to the needs of these individuals, and promoting communication with health professionals. Also, the recording, collection, and centralization of data regarding the use of mobile devices in the management of PAD can bring information that can be used for the further development of technological solutions.

Considering that the PAD population consists of elderly people with multiple comorbidities that contribute to affecting both their functional and psycho-emotional statuses, future therapeutic approaches should be individualized per patient. Health experts should take into account the particularities of PAD people, such as age, gender, educational level, basic functional capacity, and associated diseases. In addition, they should emphasize within cognitive–behavioral interventions the importance of physical exercises, adherence to treatment, and understanding the pathology to increase the motivation and self-care ability of patients. This may prove to be beneficial in optimizing therapy outcomes and reducing the risk of complications and mortality.

The conflicting results regarding the effectiveness of exercise therapy at home on inflammatory markers and quality of life require further studies in order to identify the factors that influence the results. It is also necessary to develop more effective strategies for implementing home-based exercise therapy in the management of PAD patients.

## Figures and Tables

**Table 1:** Improving functional capacity.

Study	Purpose	Parameters	Number of Patients	Follow-Up Period	Outcome	Values
Tew et al., 2015 [22]	Evaluating the improvement in functional capacity and quality of life after carrying out a group education program to promote walking in people with intermittent claudication	6-MWD, WIQ	14 patients in the intervention group 9 patients in the control group	6 weeks	Compared to the controls, the intervention group recorded improvements in the 6-MWD and the WIQ score	6-MWD: +22.9 in the intervention group vs. −20.7 in the control group. WIQ speed score: +8.7 in the intervention group vs. −3.6 in the control group. WIQ distance score: +12.5 in the intervention group vs. −0.9 in the control group. WIQ stair climbing score: +12.5 in the intervention group vs. −0.9 in the control group.
Mays et al., 2015 [28]	Efficacy of a community-based walking exercise program with training, monitoring, and coaching components to improve exercise performance and patient-reported outcomes in PAD patients	COT, WIQ	10 patients in the intervention group 10 patients in the control group	14 weeks	Changes in COT and WIQ scores were greater for intervention patients compared with control patients	COT: +1.6 in the intervention group vs. −0.6 in the control group WIQ: +18.3 in the intervention group vs. −4.6 in the control group.
Gardner et al., 2014 [29]	Comparing changes in COT and PWT in patients with symptomatic PAD following new exercise training using a home exercise program, a supervised exercise program, and a control group	PWT, COT	60 patients in the home exercise group 60 patients in the supervised exercise group 60 patients in the control group	12 weeks	Both the home walking program and the supervised exercise program demonstrated a significant increase from baseline in COT and PWT	PWT: +110 in the home exercise group vs. +192 in the supervised exercise group vs. +22 in the control group. COT: +104 in the home exercise group vs. +170 in the supervised exercise group vs. +17 in the control group.
Gardner et al., 2015 [30]	The influence of initial clinical characteristics and the duration and intensity of ambulation during a step-by-step monitored home exercise program on changes in ambulatory outcomes in symptomatic patients	PWT, COT, 6-MWD	22 men 24 women	3 months	Both men and women showed improvements in PWT and 6-MWD following the home exercise program, but COT improved statistically significantly only in men	PWT: +392 in men and +255 in women. 6-MWD: +416 in men and +267 in women. COT: +158 in men and +136 in women.
Manfredini et al., 2019 [31]	Retrospective evaluation of the association between rehabilitation outcomes and risk of peripheral revascularizations in elderly PAD patients with claudication	S_max_	743 men 264 women	The exercise program ended when no more improvements in functional parameters were registered in two consecutive tests.	S_max_ improved significantly at the end of the program both in the group of patients with moderate PAD and in those with severe PAD	S_max_: +0.4 in men and +0.4 in women.
Lamberti et al., 2020 [32]	The association between changes in exercise capacity at discharge from a home-based exercise program and the risk of all-cause mortality among patients with peripheral PAD and IC	S_max_	865 patients who completed the program 221 patients who left the program	The exercise program ended when a satisfactory and/or stable improvement in pain-free walking distance was attained.	Those who completed the program showed significant improvements in S_max_ at discharge	S_max_: +0.5 in the group who completed the program.
McDermott et al., 2021 [33]	The effectiveness of low-intensity home walking exercise at a comfortable pace on walking ability in people with PAD vs. high-intensity home walking exercise inducing ischemic leg symptoms and a no-exercise control	6-MWD	116 patients in in the low-intensity home exercise group 124 patients in the high-intensity home exercise group 65 patients in the control group	12 months	Low-intensity home exercise was significantly less effective than high-intensity home exercise and was not significantly different from the no-exercise control for improving 6-MWD	6-MWD: − 6.4 in the low-intensity home exercise group vs. +34.5 in the high-intensity home exercise group vs. − 15.1 in the no-exercise control group.
McDermott et al., 2014 [34]	Effects on functional capacity among patients who followed a program based on home walking exercises and group-mediated cognitive–behavioral therapy	6-MWD, WIQ	97 patients in the intervention group 97 patients in the control group	12 months	Following this program, 6-MWD and WIQ scores improved significantly in participants in the mediated cognitive–behavioral intervention compared to those in the other group	6-MWD: +26.5 in the intervention group vs. −7.5 in the control group WIQ: +10.4 in the intervention group vs. +1.6 in the control group.
Prévost et al., 2015 [35]	Measuring the beneficial effects of an educational therapeutic program for PAD patients	SF-36	46 patients	3 months 6 months 12 months	The results demonstrated a significant improvement in the quality of life assessed by the SF-36 score in the first three months of coaching and the long-term persistence of the results even when the patients were no longer coached	SF-36 physical composite score at 3 months + 40.8, at 6 months + 41.9 and at 12 months + 42.3 SF-36 mental composite score at 3 months + 45.2, at 6 months + 44.7 and at 12 months + 44.2.
McDermott et al., 2013 [36]	The effectiveness of a home walking exercise program based on a group-mediated cognitive–behavioral intervention on improving functional performance compared to a program based only on health education in PAD patients with and without intermittent claudication	WIQ	97 patients in the intervention group 97 patients in the control group	6 months	Home walking exercise program that used group-mediated cognitive–behavioral therapy significantly improved the WIQ score in the intervention group compared to the control group	WIQ speed score: +11.6 in the intervention group vs. +1.3 in the control group. WIQ distance score: +12.1 in the intervention group vs. +1.1 in the control group.

PAD—peripheral artery disease, 6-MWD—6 min walk distance (meters), WIQ—Walking Impairment Questionnaire, SF-36—Medical Outcomes Short Form, S_max_—maximal walking speed (kilometers/hour), COT—claudication onset time (minutes), PWT—peak walking time (minutes), IC—intermittent claudication.

## Data Availability

Not applicable.
